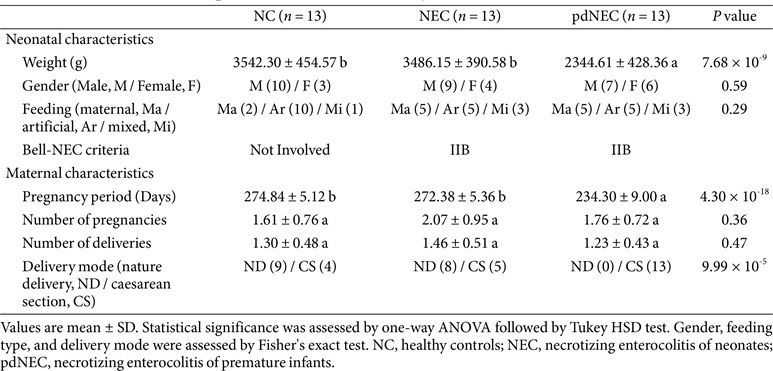# Rare and Intermediate Taxa Shape the Gut Bacterial Structure in Neonates and Preterm Infants with Necrotizing Enterocolitis

**DOI:** 10.4014/jmb.2501.01035

**Published:** 2025-05-15

**Authors:** Yu Wang, Qi Wang, Fengan Jia, Dan Li, Xuyang Gao, Xiaoge Zhang, Fan Chang, Yun Xie

**Affiliations:** 1 Department of Neonatology, Northwest Women's and Children's Hospital, Xi'an, Shaanxi 710061, P.R. China; 2 Department of Clinical Laboratory, Second Affiliated Hospital of Xi'an Jiaotong University, Xi'an, Shaanxi 710004, P.R. China; 3 Shaanxi Institute of Microbiology, Xi'an, Shaanxi 710043, P.R. China; 4 Department of Laboratory Medicine, Northwest Women's and Children's Hospital, Xi'an, Shaanxi 710061, P.R. China; 5 Medical College, Xi'an Peihua University, Xi'an, Shaanxi 710125, P.R. China; 6 Department of Pediatrics, Northwest Women's and Children's Hospital, Xi'an, Shaanxi 710061, P.R. China

**Keywords:** Necrotizing enterocolitis, premature infants, gut microbiota, association network, biomarkers

## Abstract

Necrotizing enterocolitis (NEC) is a common neonatal gastrointestinal disease with high morbidity and mortality, especially in premature infants. In a prospective case-control study, we aimed to investigate the dynamic changes in the gut microbiota of preterm infants with NEC. Infants diagnosed with NEC and preterm neonates were enrolled in this study, while normal neonates were selected as the control group. The collected samples were divided into three groups: the control group (NC), the neonatal NEC group (NEC), and the premature delivery NEC group (pdNEC). Along with basic clinical data, fecal samples from the infants (*n* = 39) were collected at the time of the first diagnosis of NEC for 16S rRNA gene sequencing. Analysis of the gut microbiota revealed no significant difference in α-diversity between infants with NEC and controls, regardless of preterm birth. The significant difference in β-diversity was primarily driven by the rare and intermediate subgroups. The rare gut subgroup found in premature infants with NEC played a crucial role in the deterministic process and specialized functionality of the microbiota, ultimately forming a sparse association network structure. Finally, multiple biomarkers of *Enterococcus* from the Firmicutes phylum were identified, providing a theoretical basis for diagnosing NEC in premature infants.

## Introduction

Necrotizing enterocolitis (NEC) is a prevalent inflammatory bowel disease in neonates, particularly affecting preterm infants [1]. The etiology of NEC is multifactorial, and involves both prenatal and postnatal factors [2]. Diagnosing its clinical manifestations is both challenging and compounded by the absence of reliable biomarkers for early detection, which hinders timely recognition and differentiation from other neonatal complications [3]. While the exact cause of NEC remains unclear, several factors have been implicated in its development, including premature birth, dysbiosis of the gut microbiota, inflammatory responses, hypoxia, blood transfusions, and suboptimal feeding practices [4, 5].

Recent advancements have significantly improved our understanding of the development of the digestive system in newborns, particularly the formation of the gut microbiota and its modulation by external factors [6, 7]. In addition to intestinal immaturity caused by premature birth, abnormal gut bacteria colonization and nutritional imbalances due to artificial feeding are major contributors to neonatal NEC [1, 6]. Previous studies have compared bacterial abundance in healthy infants and NEC patients, but most have focused primarily on changes in the dominant microbiota during NEC development. Moreover, these studies have not led to consistent conclusions [8-10].

Unlike previous studies, we conducted this prospective case-control study to examine changes in different subsets of gut microbiota in newborns and preterm infants with NEC compared to healthy controls. Our aim in this study was not only to investigate the abundance, composition, and distribution of dominant gut bacteria in infants with NEC, but also to observe changes in the intermediate and rare subpopulations of gut microbiota in preterm infants. The findings will provide valuable scientific data to inform the clinical management of NEC in pediatric practice.

## Materials and Methods

### Study Patient Cohort and Sample Collection

This study was approved by the Ethics Committee of Northwest Women's and Children's Hospital (2022-017). A total of 39 infants (0 to 1 months) attending Northwest Women's and Children's Hospital were enrolled from May 2023 to April 2024 and included healthy infants as negative control cases (NC, 13 cases), a necrotizing enterocolitis group (NEC, 13 cases), and a premature delivery NEC group (pdNEC, 13 cases). All participants were from the same children's ward. Infants were excluded if they had received antibiotics or probiotics or had a known history of any other diseases. Clinical data for each infant, including weight, gender, feeding habits, and Bell-NEC criteria, were recorded. Clinical data for the postpartum women, including gestational period, number of pregnancies, number of deliveries, and mode of delivery, were also documented. Fecal samples from all infants were collected using a validated stool collector, processed with liquid nitrogen three times, and stored at −80°C until further analysis [11].

### DNA Extraction, Amplification, and Sequencing

DNA was extracted from different samples using the E.Z.N.A. Stool DNA Kit (D4015, Omega Bio-Tek, USA) according to the manufacturer’s instructions. This reagent, specifically designed to detect even trace amounts of DNA, is highly effective in extracting DNA from most bacterial species. In the DNA extraction process, a blank control performed with nuclease-free water was included to monitor potential reagent-derived contamination. The blank control was subjected to the same downstream PCR and sequencing workflows as the experimental samples. The elution buffer was used to extract total DNA, which was stored at −80°C until further analysis by PCR, conducted by LC-Bio Technology Co. Ltd., China.

The V3-V4 region of the prokaryotic small-subunit (16S) rRNA gene was amplified using primers 341F (5'-CCTACGGGNGGCWGCAG-3') and 805R (5'-GACTACHVGGGTATCTAATCC-3') [12]. Each primer was tagged with a unique barcode for the corresponding sample, and sequencing universal primers were added to their 5' ends. PCR amplification was performed in a 25 μl reaction mixture, which included 25 ng of template DNA, 12.5 μl of PCR Premix, 2.5 μl of each primer, and PCR-grade water to adjust the volume. The PCR conditions consisted of an initial denaturation at 98°C for 30 sec, followed by 32 cycles of denaturation at 98°C for 10 sec, annealing at 54°C for 30 sec, and extension at 72°C for 45 sec. A final extension step was performed at 72°C for 10 min. The PCR products were verified by 2% agarose gel electrophoresis. To ensure accurate PCR results and avoid contamination, ultrapure water was used as a negative control in place of the sample solution. The PCR products were purified using AMPure XT beads (Beckman Coulter Genomics, USA) and quantified with Qubit (Invitrogen, USA). Amplicon pools were prepared for sequencing using the Agilent 2100 Bioanalyzer (Agilent, USA), and the size and quantity of the amplicon library were assessed with the Library Quantification Kit for Illumina (Kapa Biosciences, USA). The libraries were sequenced on the NovaSeq6000 platform. Raw data from 39 samples are available from the Sequence Read Archive (SRA) under accession number PRJNA1180186.

### Sequence Processing and Taxonomic Affiliation

The samples were sequenced on an Illumina NovaSeq6000 platform following standard protocols. Paired-end reads were assigned to each sample based on their unique barcode, then trimmed to remove the barcode and primer sequences. Data analysis was performed using the Usearch10 (https://www.drive5.com/usearch/manual10/) pipeline [13] to process the 16S rRNA data. The forward and reverse reads were merged, assigned to samples by barcode, and trimmed to eliminate the barcode and primer sequences. The quality filtering process applied to the merged sequences included ensuring the absence of ambiguous bases, maintaining an expected error rate per base below 0.01, performing dereplication, and eliminating singleton sequences with a size of less than 8. Sequences were grouped into amplicon sequence variants (ASVs) using the Unoise3 algorithm [14, 15], with chimeric sequences removed. Sequences were classified with the Vsearch 2.8.1 program against the Ribosomal Database Project v11.4 (RDP, http://rdp.cme.msu.edu/) database and taxonomy assignment was performed using the RDP classifier [16] at a confidence threshold of 0.8.

### Data Analysis

Statistical analyses were performed using R software (v4.4.1; http://www.r-project.org/). Ecological differences in subcommunities among infants with NEC were assessed by classifying ASVs into three subgroups based on relative abundance: rare taxa (those with a relative abundance of ≤ 0.01% in one sample), intermediate taxa (those with a relative abundance of 0.01%–0.1% in one sample), and abundant taxa (those with a relative abundance of ≥ 0.1% in one sample) [17-19]. α-diversity was calculated using Richness, Shannon, and Pielou’s evenness indices with the “vegan” package (version 2.5-6) [20]. Phylogenetic analysis was performed using the Usearch10 cluster_agg method [13] with Bray-Curtis distances. α-diversity indices were compared using one-way ANOVA and Tukey's test. Principal coordinate analysis (PCoA) was performed using Bray-Curtis dissimilarities, and PERMANOVA was conducted with the adonis function [20].

The “NST” package (version 2.0.4) was used to calculate the taxonomic normalized stochasticity ratio (tNST) to assess the role of stochasticity in shaping community structure [21]. The assembly processes of bacterial communities were evaluated by calculating the beta nearest taxon index (βNTI) using the NST package. The standardized effect size (SES) approach was employed to compare observed phylogenetic turnover against null expectations [21]. When |βNTI| < 2, the contribution was considered a stochastic process, and when |βNTI| > 2, the shifts in community were deterministic processes. Also, the combination matrix of βNTI values and Bray-Curtis-based Raup-Crick (RC bray) was applied to estimate the relative contributions of homogeneous selection, heterogeneous selection, dispersal limitation, homogenizing dispersal, and drift processes in governing community assembly. βNTI of < −2 or > 2 indicated homogeneous selection or heterogeneous selection, respectively. |RC bray| > 0.95 indicated significant deviations from the null model expectation. |βNTI| of < 2 with RC bray <-0.95 or > 0.95 indicated a contribution of homogenizing dispersal or dispersal limitation, respectively. Otherwise (|βNTI| ≤ 2 and |RC bray| ≤ 0.95), the shifts in community were drift. The significance of the tNST and βNTI was assessed by ANOVA tests [22]. The niche breadth index was calculated using Levin’s equation with the spaa package (version 0.2.1) [23]. ASVs were classified as SPECIALIST, GENERALIST, or OPPORTUNIST based on their niche breadth index: SPECIALIST if below the lower limit, GENERALIST if above the upper limit, and OPPORTUNIST if within the 95% confidence interval [24].

Taxonomic analysis was performed using the "ggplot2" package (version 3.3.6) to create stacked bar plots at the phylum and genus levels, showing species abundance. An association network was constructed based on Spearman’s correlation coefficients, with a 0.6 cutoff, including only significant edges (*p* < 0.05). Topological properties were calculated using Gephi (v0.10; https://gephi.org), and relative abundance of ASVs was displayed in Gephi at a ratio of 10 to 200 times.

Random forest models were built using the "random Forest" package (version 4.6-14) [25]. Nested cross-validation identified key biomarkers. The mean decrease in accuracy, Gini, and relative abundance of biomarkers were used to assess their importance across groups.

Comparisons among multiple groups were analyzed using one-way analysis of variance (ANOVA), and pairwise comparisons after ANOVA were conducted by Tukey’s multiple comparisons test. *P* values were obtained using a two-tailed *t*-test, and *p* < 0.05 indicated a significant difference. The results were presented as mean±SD.

## Results

### Study Population Characteristics

A total of 13 neonates with necrotizing enterocolitis (NEC), 13 premature delivery infants with NEC (pdNEC), and 13 healthy controls (NC) were enrolled. The pdNEC group had significantly different pregnancy duration and birth weight compared to the other groups, likely due to preterm birth, and all preterm infants were delivered by cesarean section. Gender, feeding patterns, and maternal factors (number of pregnancies and deliveries) were not significantly different between groups to minimize potential confounding effects on the microbiota (Table 1). PCR amplification of blank control (nuclease-free water processed in parallel) showed no detectable bands on agarose gel electrophoresis, confirming the absence of exogenous DNA contamination from reagents or laboratory environments.

### Alpha and Beta Diversity of Bacterial Communities Influenced by Intermediate and Rare Subgroups

After filtering and removing chimeras, 39 samples yielded 2,696,395 high-quality sequences (mean: 69,138 per sample), which were clustered into 1,175 bacterial ASVs using Unoise3.

**Fig. 1:**
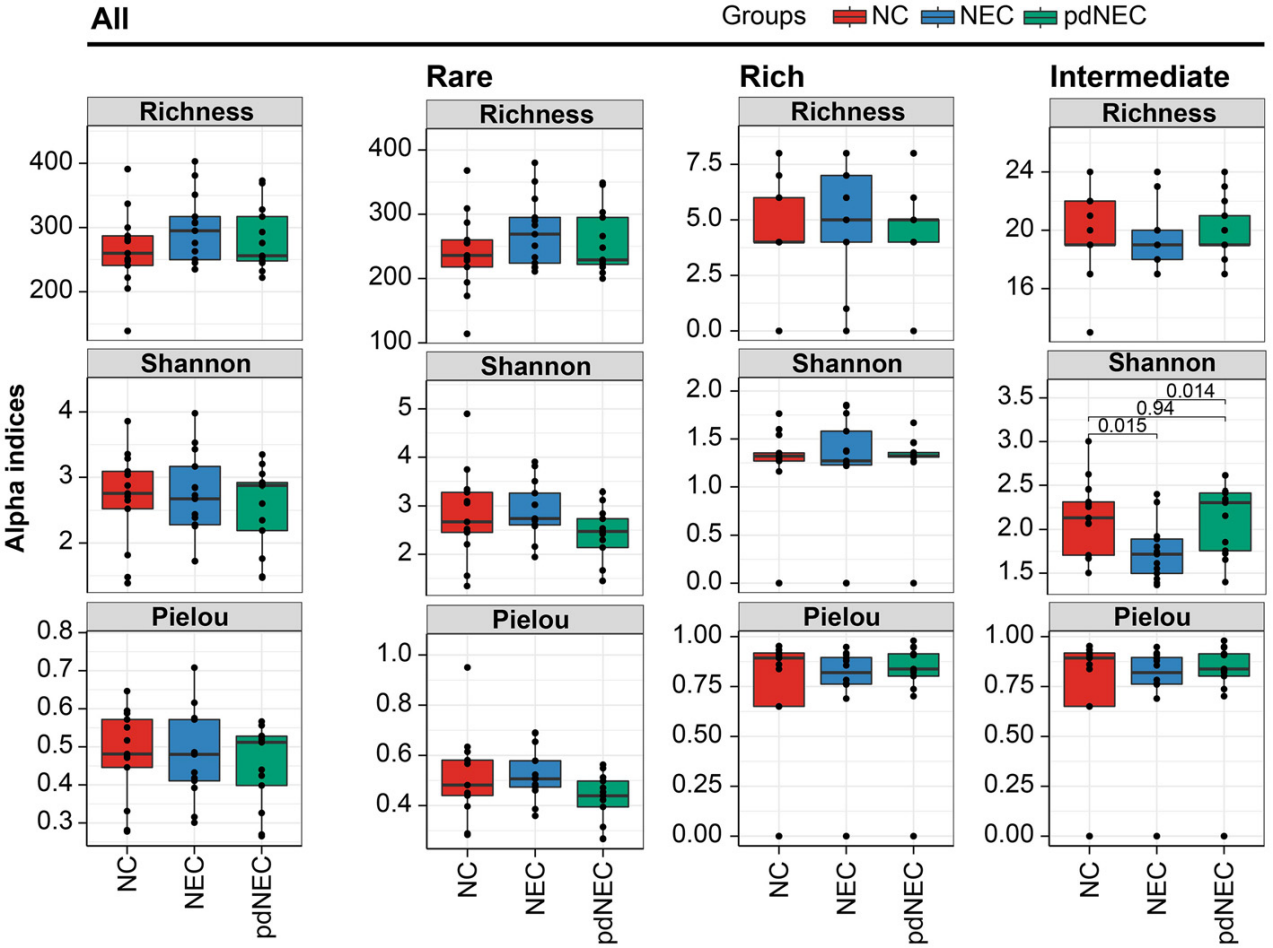
Comparison of intestinal microbiota structure at the amplicon sequence variant (ASV) level in NC, NEC, and pdNEC. The ASVs were classified into rare (≤ 0.01%), rich (≥ 0.1%), and intermediate (between 0.01% and 0.1%) subgroups based on their relative abundance, and the corresponding alpha diversity indices were calculated. ANOVA and Tukey’s multiple comparison tests were used for comparisons and different lowercase letters represented significant differences.

The α-diversity of bacterial communities of neonates and preterm infants with necrotizing enterocolitis (NEC) was assessed. While there was no significant difference overall, we observed an increase in the median richness index in the NEC group and a decrease in pdNEC group compared to the NCs. Interestingly, the Shannon and Pielou indices displayed an opposite trend (Fig. 1).

Distinct α-diversity trends emerged across subgroups. The NEC group had the highest Richness and Shannon indices, while the intermediate subgroup had the lowest. In the rich subgroup, the NEC group also showed the highest Richness and Shannon indices, opposite to the intermediate subgroup. Notably, the trend among the Richness index of the rare subgroup was consistent with that of bacteria microbiota, suggesting that richness was driven by rare bacterial taxa.

**Fig. 2:**
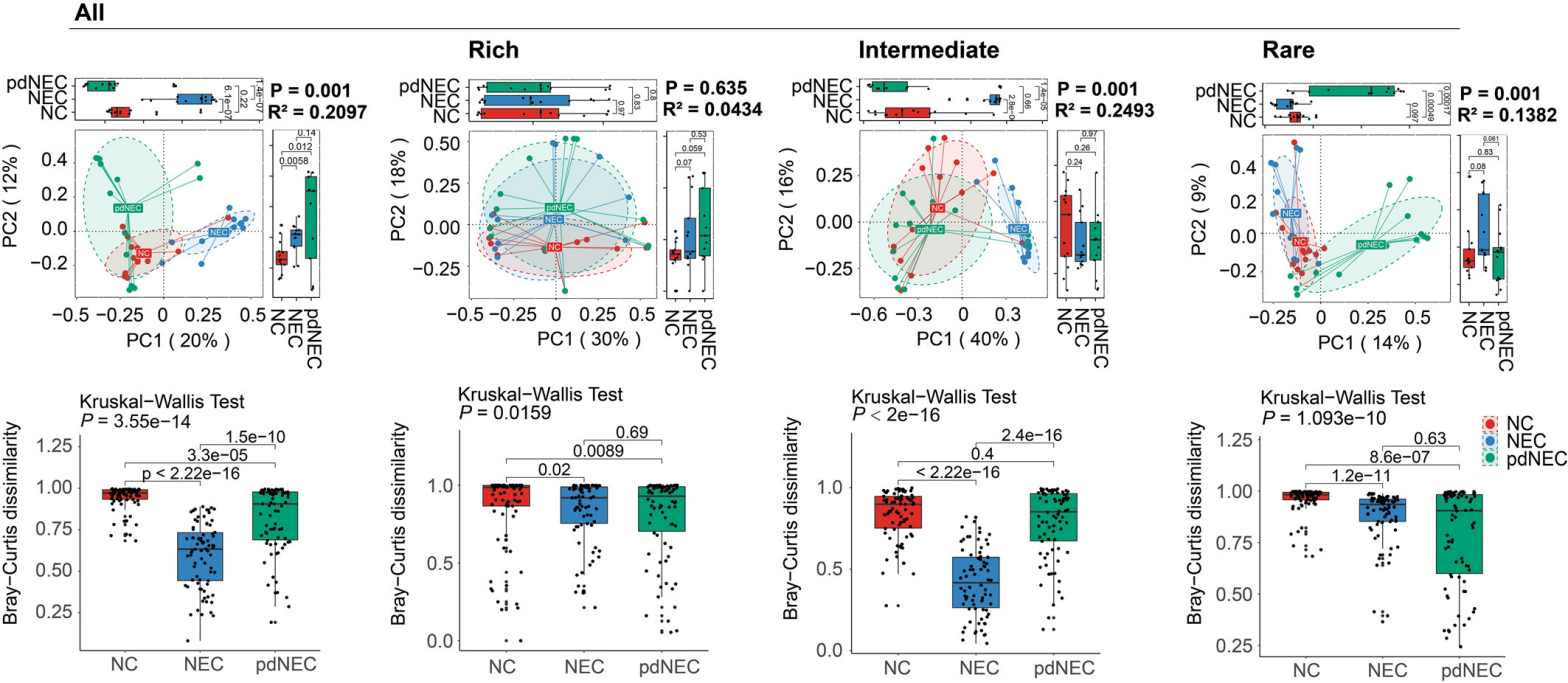
Comparisons to the β-diversity distance and dissimilarity index of intestinal microbiota in different groups (NC, NEC, and pdNEC). Significant changes in beta diversity were calculated using PERMANOVA on the Bray-Curtis distance matrix. Different lowercase letter represented significant differences of dissimilarity index between groups, based on Kruskal-Wallis test combined Wilcoxon tests.

To analyze variations in β-diversity of bacterial communities, the Bray-Curtis dissimilarity matrix was used. PCoA revealed distinct microbiota structures in both the NEC and pdNEC groups compared to the healthy controls. Collectively, PCoA explained 32% of the variation in bacterial community composition (PC1, 20%; PC2, 12%), and the delivery mode of the infants did not significantly affect the variation (Fig. S1). The β-diversity of the NEC group clustered distinctly along the PC1 axis, while that of the pdNEC group was separately clustered on the PC2 axis. PERMANOVA confirmed significant differences in bacterial community structure between the NC, NEC, and pdNEC groups (*p* = 0.001). Additionally, statistically significant variations were observed in the dissimilarity index across groups. The NEC group exhibited the lowest dissimilarity index, followed by the pdNEC group. The β-diversity of the pdNEC group clustered distinctly along the PC1 axis in the rare subgroup (*p* = 0.001), while the NEC group showed a significant difference in the intermediate subgroup (*p* = 0.001). In contrast, no significant differences were observed in the rich subgroup. These results suggest that differences in β-diversity within the NEC group may be driven by the intermediate subgroup, whereas variations in the pdNEC group are likely attributed to the rare subgroup. The Bray-Curtis dissimilarity index for the intermediate subgroup followed the overall bacterial trend, while the NC group had significantly higher indices in both the rare and rich subgroups compared to the others (Fig. 2).

### Assembly Processes and Changes in Niche Breadth of Bacterial Gut Bacterial Communities

**Fig. 3:**
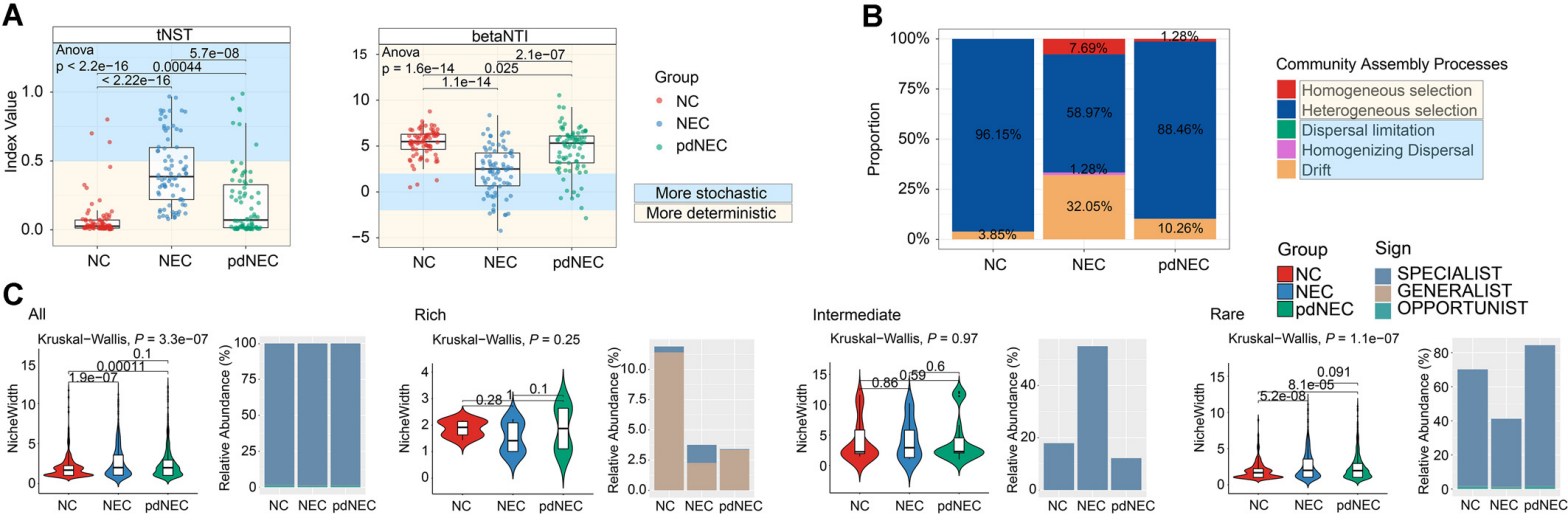
Comparison of tNST, βNTI, and niche breadth of intestinal microbiota in NC, NEC, and pdNEC groups. (**A**) tNST values for bacterial communities for NC, NEC, and pdNEC groups. Light yellow and light blue background color indicated tNST thresholds of 0.5. (**B**) βNTI values for bacterial communities for different groups. Light-yellow and light-blue background color indicated betaNTI thresholds of +2 and −2. (**C**) Proportion of community assembly processes in different groups, and the proportion of 0.00% is deleted. Significant changes in tNST, βNTI, and niche breadth were calculated using one-way ANOVA followed by Tukey HSD tests, and significant differences were indicated by horizontal bars with corresponding *p*-values.

To quantify the roles of deterministic and stochastic processes in gut bacterial community succession, tNST and βNTI were calculated. Overall, deterministic processes dominated the gut bacterial communities of all groups (tNST median < 0.5), and the communities of the NEC group exhibited a shift toward stochasticity, with an average tNST of 0.43 (Fig. 3A, Table S1). Additionally, the βNTI results further confirmed that deterministic processes (βNTI median > 2) dominated the assemblages of the bacterial communities (Fig. 3B, Table S1). In terms of the proportion of community assembly process, heterogeneous selection was the highest in the NC group (96.15%), followed by the pdNEC group (88.46%). Homogeneous selection (7.69%), homogenizing dispersal (1.28%) and drift (32.05%) occupied the highest proportion in the NEC group. In addition, there was no homogeneous selection and homogenizing dispersal in the NC group and no homogenizing dispersal in the pdNEC group. Moreover, the dispersal limitation was not observed in any group (Fig. 3C). The niche breadth of bacterial communities in the NEC and pdNEC groups was significantly higher than in the NC group (average niche breadth: NC, 1.99; NEC, 2.65; pdNEC, 2.45), primarily due to the rare subgroup (average niche breadth: NC, 1.91; NEC, 1.87; pdNEC, 2.40; Fig. 3 and Table S2). The intermediate subgroup had a broader niche breadth (average: NC, 3.96; NEC, 4.27; pdNEC, 4.06), while the rich subgroup had a narrower breadth (average: NC, 1.87; NEC, 1.53; pdNEC, 1.87). The rare subgroup was dominated by SPECIALIST species (relative abundance: NC, 68.40%; NEC, 39.92%; pdNEC, 82.58%), while OPPORTUNIST species were more common in the rich subgroup (relative abundance: NC, 11.41%; NEC, 2.27%; pdNEC, 3.36%) (Table S2). Interestingly, we found that GENERALIST ASVs in the rich subgroup were not observed in any of the three groups. These findings suggest that the majority of SPECIALIST species, with high relative abundance of ASVs, are primarily found in rare subgroups, indicating their significant role in community succession and functional development.

### Taxonomic Compositions and Subgroup Distribution of Gut Bacteria in Infants with NEC

**Fig. 4:**
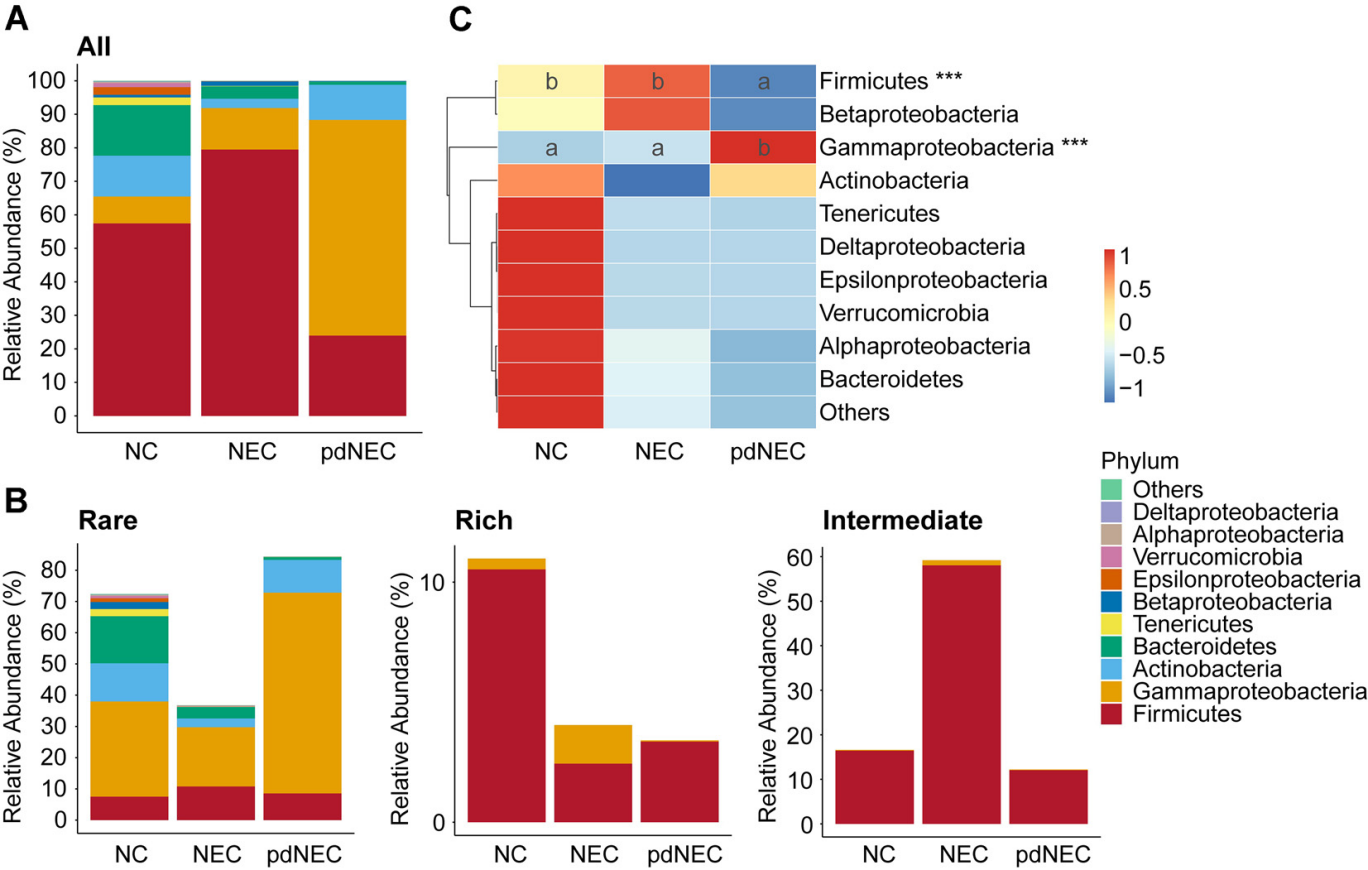
Relative abundance of the bacterial phyla and the distribution of species of different subgroups in each clinical group. (**A**) Relative abundance of ASVs at phylum level between groups. (**B**) Distribution of phyla in different subgroups. (**C**) Clustering heatmap of ASVs at the phylum level.

The composition and relative abundance of bacterial communities at the phylum level are shown in Fig. 4A, 4C, and Table S3. Firmicutes and *Gammaproteobacteria* were dominant across all groups. The average relative abundance of Firmicutes decreased significantly in the pdNEC group (NC, 57.453%; NEC, 79.475%; pdNEC, 23.980%; *p* < 0.001), while *Gammaproteobacteria* increased (NC, 8.013%; NEC, 12.389%; pdNEC, 64.344%; *p* < 0.001). The NC group exhibited higher abundances of Actinobacteria and Bacteroidetes, with Actinobacteria also more abundant in the pdNEC group. However, no significant differences in species composition were observed between the groups.

Firmicutes dominated the rich and intermediate subgroups, while *Gammaproteobacteria* were predominantly found in the rare subgroup. Additionally, other phyla, including Actinobacteria, Bacteroidetes, and Tenericutes, were identified in the rare subgroup (Fig. 4B).

### Bacterial Microbiota Association Network in Infants of NEC

**Fig. 5:**
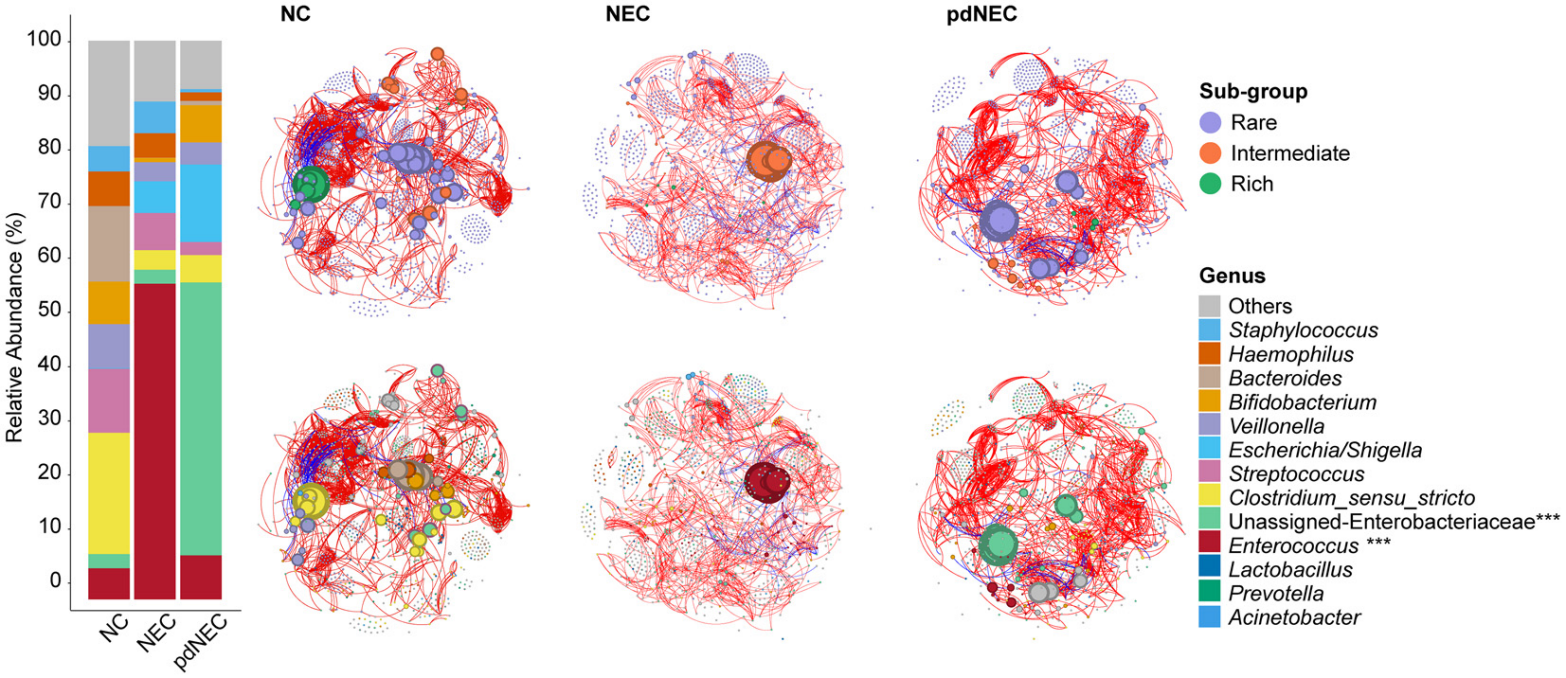
Relative abundance and association networks of the intestinal genera in different NEC groups. Spearman rank correlation analysis was performed to calculate the correlation coefficient and displayed ASVs with a correlation >0.6 and a *p*-value of <0.05. The red side indicated a positive correlation, and the blue side represented a negative correlation. The size of the nodes reflected the average relative abundance of ASVs, and nodes belonging to a cluster had the same color.

Association networks were established to analyze the structure and interrelationships of bacterial communities across different groups. At the genus level, dominant species differed between the groups. *Clostridium sensu stricto*, *Streptococcus*, and *Bacteroides* were more abundant in the NC group, while *Enterococcus* dominated in the NEC group. In the pdNEC group, *Escherichia/Shigella*, *Bifidobacterium*, and *Enterococcus* were predominant, with numerous Unassigned-Enterobacteriaceae ASVs also detected (Fig. 5). The more correlated ASVs (Spearman’s correlation coefficient > 0.6 and *p* < 0.05) also showed distinct patterns across the groups. The NC group exhibited high abundance in both rich and rare subgroups, mainly consisting of *Clostridium sensu stricto*, *Bacteroides*, *Bifidobacterium*, and *Haemophilus*. *Enterococcus* was abundant in the intermediate subgroup of the NEC group, while high-abundance ASVs in the pdNEC group were primarily from rare subgroups, mainly Unassigned-Enterobacteriaceae and Others with lower abundance. Correlations were observed between *Enterococcus*, *Bifidobacterium*, and *Clostridium sensu stricto*, all of which were abundant.

In terms of network connectivity and complexity, the NC group had a similar number of nodes (NC, 1153; NEC, 1119; pdNEC, 1093), but about 100 more edges than the NEC and pdNEC groups (NC, 2458; NEC, 1453; pdNEC, 1572). Additionally, the NC group exhibited the highest average degree distribution (66.81), reflecting superior network connectivity (Fig. 5, Table S4). The NEC and pdNEC groups exhibited higher modularity (NC, 0.506; NEC, 0.512; pdNEC, 0.518) and longer average path lengths (NC, 2.585; NEC, 2.617; pdNEC, 2.603), indicating dense interconnections within each community and a more complex network. Notably, despite having the highest modularity, the pdNEC group had the fewest modules (NC, 7; NEC, 8; pdNEC, 6), suggesting dense intra-module connections but sparse inter-module connections (Fig. 5).

### Identification and Distribution of Biomarkers for NEC Diseases Based on Bacterial ASVs

**Fig. 6:**
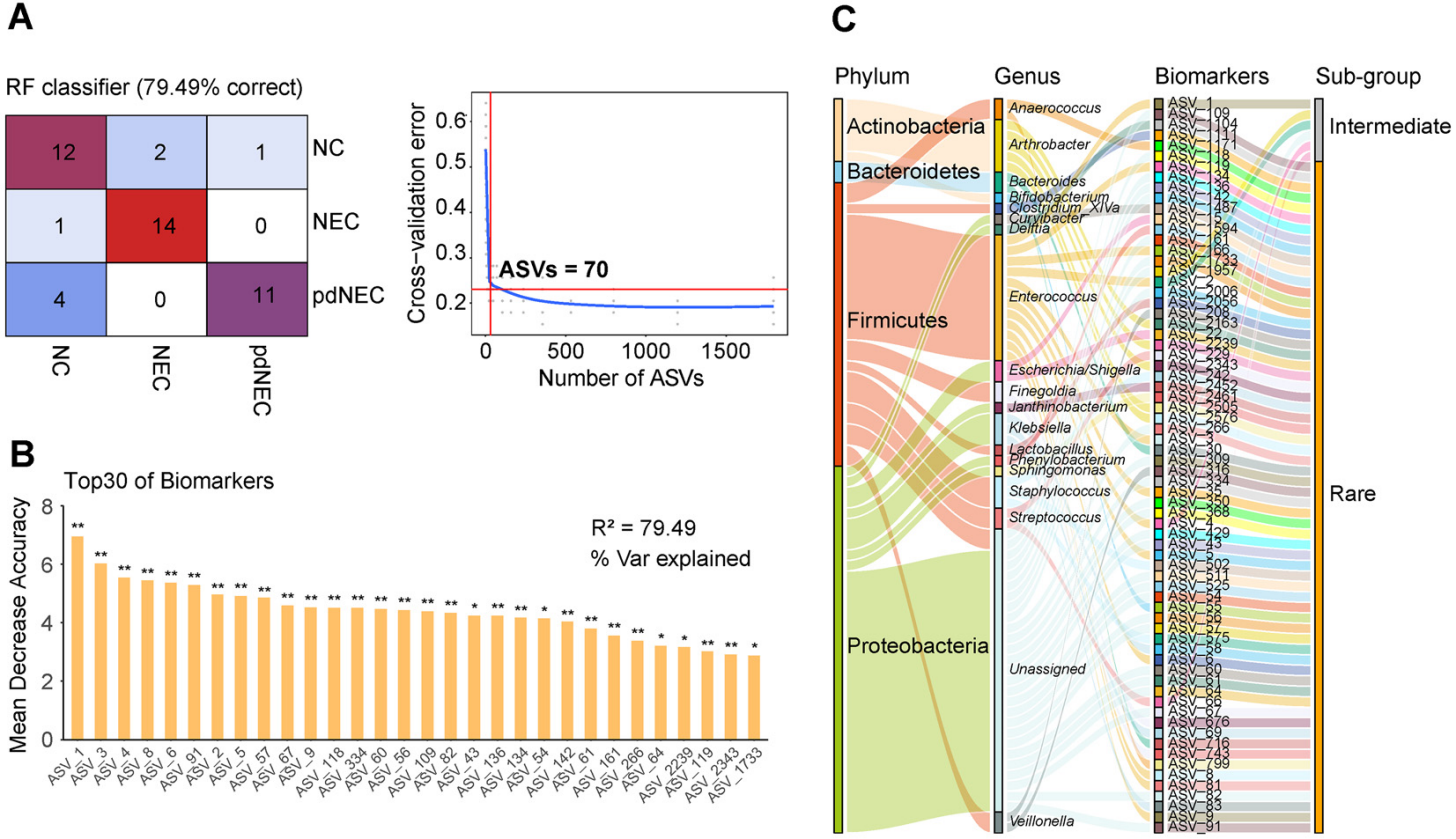
Identification of bacterial biomarkers of NECs by random forest models. (**A**) A 10-fold cross-validation on a random forest model was performed to detect unique amplicon sequence variant (ASV)-based markers. (**B**) The mean decreased accuracy and importance of the top 30 biomarkers. (**C**) Relative abundance distribution of biomarkers at phyla level, genus level, and different subgroups.

To evaluate the ability of gut bacterial biomarkers to classify neonates and preterm infants with NEC, a random forest classifier (RFC) model was developed. A 10-fold cross-validation was conducted to identify unique ASV-based biomarkers. The analysis revealed the top 70 differentially abundant markers as the optimal biomarker set. Using these 70 markers, the RFC model achieved an accuracy of 79.49% (Fig. 6A). Additionally, the top 30 biomarkers significantly contributed to the model, highlighting their importance and accuracy (Fig. 6B).

The bacterial biomarkers consisted of four phyla: Firmicutes (23.58%), Proteobacteria (12.06%), Bacteroidetes (1.49%), and Actinobacteria (0.88%). Proteobacteria had the highest number of ASVs (35), followed by Firmicutes (27), Actinobacteria (6), and Bacteroidetes (2) (Fig. 6C, Table S4). At the genus level, *Enterococcus* had the most ASVs (12) and the highest abundance (21.652%). Of the 70 biomarkers, 64 were from the rare subgroup, 6 from the intermediate subgroup, and none from the rich subgroup. The top five ASVs by Mean Decrease Accuracy-ASV_1, ASV_3, ASV_4, ASV_8, and ASV_6-were all Enterococcus. ASV_1, ASV_3, and ASV_4 were in the intermediate subgroup, while ASV_8 and ASV_6 were in the rare subgroup (Fig. 6B). These findings suggest that the intermediate and rare subgroups, particularly *Enterococcus*, play a crucial role in classifying NEC. Notably, a significant abundance of Unassigned-Enterobacteriaceae biomarkers associated with Proteobacteria (12.01%) indicates the presence of numerous unidentified species in NEC that remain to be explored.

## Discussion

### Rare Species Determined α-Diversity and Intermediate Species Determined β-Diversity in the Gut Microbiota of Infants with NEC

Preterm birth, antibiotic use, and artificial feeding are significant factors contributing to neonatal NEC [2]. In this study, we focused on the impact of preterm birth on NEC in neonates, excluding other neonatal and maternal factors (Table 1). Previous studies have shown that NEC reduces bacterial diversity [26, 27], while more recent research found no significant differences in gut bacterial diversity between infants with NEC and healthy newborns [28]. Our results showed an increase in the richness and a decrease in the diversity of bacterial communities in infants and premature delivery with NEC compared to newborns, although these differences were not statistically significant (Fig. 1). Additionally, we characterized the gut microbiota structure and dynamics in both newborns and preterm infants with NEC. A consistent trend was observed between α-diversity changes and those in the rare subgroup, highlighting the critical role of rare ASVs in shaping the gut microbiota during neonatal NEC.

Our results showed significant β-diversity differences in gut microbiota between healthy newborns, NEC newborns, and NEC preterm infants, consistent with previous studies [29, 30]. Bray-Curtis dissimilarity also varied significantly among groups, primarily influenced by rare and intermediate subgroup taxa (Fig. 2). A core community of low-abundance bacteria, as shown in prior studies [31, 32], comprised a large portion of the microbiome. These findings suggest that preterm birth-related dysbiosis disrupts the microbiota structure in infants with NEC, further impairing gut microecology. This disruption might come from the unsaturated and dynamic state of the microbiota in premature infants [33].

### In Different NEC Diseases, Different Subpopulations of Bacterial Communities Exhibited Different Disparate Assembly Strategies and Differing Ecological Functions

The neonatal gut microbiome might be strongly influenced by gestational age and is associated with both short-term growth and long-term health outcomes [34]. For preterm infants, gut microbiota health is shaped by environmental factors, host interactions, and microbial dynamics [35]. The preterm microbiota was crucial to gut health and might contribute to NEC, the most significant pathology affecting preterm infants [36]. In our study, we observed that NEC disturbed the assembly strategies of the gut bacterial communities of newborns and preterm infants, with an increased proportion of stochastic processes (Fig. 3).

Species with wider niche breadth are GENERALISTS, less influenced by environmental factors due to higher tolerance [37]. We found that the niche breadth of gut bacteria increased proportionally with their abundance (Fig. 3), suggesting that species in rich subgroups were less affected by gut disturbances, while species in rare subgroups exhibited faster succession, as seen in the Bray-Curtis dissimilarity (Fig. 2). The rich subgroup was mainly composed of GENERALIST species, with the highest abundance in the gut of healthy newborns. In contrast, SPECIALIST species were predominantly part of the rare subgroup, particularly in preterm infants with NEC. These findings suggest that preterm birth disrupts gut microecology, increasing bacteria with specialized functions.

### Differences in Taxa Clusters in Different Subgroups Affected Gut Microbiome Association Networks in NEC Newborns and Preterm Infants

In this study, Proteobacteria were classified to understand their distribution patterns. Results showed that *Gammaproteobacteria* were significantly increased and Firmicutes were significantly decreased in preterm infants with NEC, with most *Gammaproteobacteria* originating from rare subpopulations. A previous study indicated that *Gammaproteobacteria* could increase or remain consistent in the intestines of very low birth-weight infants, potentially reducing ATP and beneficial SCFA production, which could increase the risk of inflammatory and immune responses in the intestine and other systems [38]. *Firmicutes* are obligate anaerobes that reduce intestinal oxidative stress [39]. Studies have found that *Firmicutes* are severely depleted in preterm infants [32].

Taxonomic differences significantly influenced the gut microbiota network characteristics. In NEC infants, *Enterococcus* was abundant, consisting of a few high-abundance ASVs from the intermediate subgroup. In contrast, the microbiota of preterm NEC infants contained high-abundance Proteobacteria species, all belonging to the rare subgroup (Figs. 5 and 6). Previous studies have shown that *Enterococcus* can prevent NEC and reduce its incidence. It plays an active role in neonatal NEC progression by fermenting carbohydrates to produce lactic acid [40], regulating T cell immunity, activating cytotoxicity [41], and supporting gut microbiota homeostasis [42]. The successful treatment of infants in the NEC group of our study suggests that the significant presence of *Enterococcus* and its central role in the network may have had a positive influence.

We found that the NEC association network in preterm infants had the fewest nodes, highest modularity, and fewest modules (Fig. 5, Table S4). Compared to the normal group, preterm infants with NEC had a sparse gut microbiota correlation network, with reduced complexity and elasticity, making the microflora more susceptible [43].

### Rare and Intermediate Biomarkers Distinguished Neonatal and Preterm NEC

Machine learning has proven effective in classifying and predicting NEC [28]. Our study demonstrated that, even with the influence of NEC and preterm birth factors, the random forest algorithm successfully identified ASV-based biomarkers and accurately differentiated between study cohorts. Seventy ASV taxa were selected as biomarkers based on RFC and cross-validation error, improving classification accuracy (Fig. 6A and 6B).

*Enterococcus* has been identified as a probiotic that helps prevent and treat NEC, demonstrating its regulatory effect on intestinal barrier damage caused by necrotic enteritis [44, 45]. In our study, the top five biomarkers based on Mean Decrease Accuracy were all *Enterococcus*, suggesting that its abundance plays a key role in the classification and diagnosis of NEC. Notably, all NEC-predicting biomarkers originated from rare and intermediate ASV subgroups (Fig. 6C). Previous studies have shown that rare taxa are phylogenetically diverse and can independently or collectively contribute to metabolic functions vital for human health, serving as reservoirs of genetic and functional diversity [31, 46].

## Conclusion

The α-diversity of gut bacterial communities was not significantly affected by NEC or preterm birth. The decline in diversity and evenness observed in NEC infants was primarily due to rare species with low relative abundance. Preterm birth significantly influenced the β-diversity of gut bacterial communities in NEC infants. The intermediate subgroup played a dominant role in clustering preterm NEC infants at the β-diversity distance and in the change in the Bray-Curtis dissimilarity index. The rare subgroup also followed a deterministic assembly strategy with a narrow niche breadth, and was dominated by specialist species, resulting in a gut flora in NEC infants with specialized functions. *Gammaproteobacteria* increased and Firmicutes decreased significantly in preterm infants with NEC, forming a sparse correlation network. *Enterococcus* was identified as the primary biomarker distinguishing NEC from preterm NEC infants.

Microbiome biomarkers are promising tools for accurately predicting NEC and guiding treatment selection. Further studies with longer follow-up periods are needed to identify potential diagnostic biomarkers for NEC.

## Supplemental Materials

Supplementary data for this paper are available on-line only at http://jmb.or.kr.



## Figures and Tables

**Table 1:** Characteristics of the patients included in the study.