# Supplementary material for: Rare and Intermediate Taxa Shape the Gut Bacterial Structure in Neonates and Preterm Infants with Necrotizing Enterocolitis
Source: J Microbiol Biotechnol. 2025 May 15;35:e2501035. doi: 10.4014/jmb.2501.01035 (PMC12099621; doi:10.4014/jmb.2501.01035)
Supplement: Supplementary file 1 [file jmb-35-e2501035-supple.pdf]

J. Microbiol. Biotechnol. 2025. 35: e2501035
https://doi.org/10.4014/jmb.2501.01035

Supplementary Tables and Figure

Table S1. tNST, βNTI and Niche breadth of the groups included in the study. All values were an average from all replicates ± standard deviations. Values with the same letters within a row did not significantly differ based on the Tukey's multiple comparisons test.

**Index**	**group**	**NC**	**NEC**	**pdNEC**	**P value**
tNST	All	0.08 ± 0.15**c**	0.43 ± 0.25**a**	0.20 ± 0.25**b**	0.03
βNTI	All	5.33 ± 1.53**a**	2.31 ± 2.62**c**	4.56 ± 2.55**b**	1.6×10^{-14}
Niche breadth	All	1.99±1.37**b**	2.65±1.94**a**	2.45±1.86**a**	3.3×10^{-7}
	Rare sub-group	1.91±1.18**b**	2.60±1.85**a**	2.40±1.72**a**	1.1×10^{-7}
	Rich sub-group	1.87±0.30	1.53±0.58	1.87±0.81	0.25
	Intermediate sub-group	3.96±3.38	4.27±3.21	4.06±3.23	0.97

Table S2. Relative abundance of different types of ASVs across niche breadth included in the study. All values were average relative abundance (%).

**Average relative abundance (%)**				
		**NC**	**NEC**	**pdNEC**
All	GENERALIST	0.26	0.31	0.21
	OPPORTUNIST	1.41	0.93	1.20
	SPECIALIST	98.32	98.77	98.60
Rare sub-group	GENERALIST	0.24	0.29	0.20
	OPPORTUNIST	1.54	1.02	1.61
	SPECIALIST	68.40	39.92	82.58
Rich sub-group	GENERALIST	0 (Not observed)	0 (Not observed)	0 (Not observed)
	OPPORTUNIST	11.41	2.27	3.36
	SPECIALIST	0.49	1.49	0.05
Intermediate sub-group	GENERALIST	0.01	0.01	0.01
	OPPORTUNIST	0.01	0.01	0.01
	SPECIALIST	17.91	54.99	12.20

Table S3. The relative abundance at phylum level of different sub-groups in each clinical group. All values were an average from all replicates ± standard deviations. Values with the same letters within a column did not significantly differ based on the ANOVA and Tukey's multiple comparisons tests.

	**Phylum**	**NC**	**NEC**	**pdNEC**	**ANOVA Pvalue**
All	**Firmicutes**	57.453 ± 38.158 **b**	79.475 ± 22.410 **b**	23.980 ± 23.916 **a**	9.94×10^{-5}
	**Gammaproteobacteria**	8.013 ± 14.081 **a**	12.389 ± 18.757 **a**	64.344 ± 25.448 **b**	1.04×10^{-8}
	Actinobacteria	12.197 ± 20.846 a	2.818 ± 3.632 a	10.496 ± 17.081 a	ns
	Bacteroidetes	15.099 ± 28.859 a	3.698 ± 13.167 a	0.890 ± 2.975 a	ns
	Tenericutes	2.260 ± 8.144 a	0.112 ± 0.402 a	0.010 ± 0.034 a	ns
	Betaproteobacteria	0.818 ± 1.286 a	1.344 ± 3.548 a	0.214 ± 0.091 a	ns
	Epsilonproteobacteria	2.291 ± 8.253 a	0.007 ± 0.021 a	0.002 ± 0.002 a	ns
	Verrucomicrobia	1.206 ± 4.340 a	0.003 ± 0.003 a	0.001 ± 0.002 a	ns
	Alphaproteobacteria	0.315 ± 0.514 a	0.111 ± 0.240 a	0.048 ± 0.036 a	ns
	Deltaproteobacteria	0.167 ± 0.601 a	0.000 ± 0.000 a	0.000 ± 0.000 a	ns
	Others	0.181 ± 0.487 a	0.044 ± 0.071 a	0.016 ± 0.015 a	ns
Rare sub-group	**Gammaproteobacteria**	7.564 ± 14.241 **a**	10.786 ± 17.198 **a**	64.294 ± 25.466 **b**	4.26×10^{-9}
	**Firmicutes**	30.470 ± 29.680 **b**	18.964 ± 17.151 **ab**	8.558 ± 5.948 **a**	0.03
	Actinobacteria	12.197 ± 20.846 a	2.818 ± 3.632 a	10.496 ± 17.081 a	ns
	Bacteroidetes	15.099 ± 28.859 a	3.698 ± 13.167 a	0.890 ± 2.975 a	ns
	Tenericutes	2.260 ± 8.144 a	0.112 ± 0.402 a	0.010 ± 0.034 a	ns
	Epsilonproteobacteria	2.291 ± 8.253 a	0.007 ± 0.021 a	0.002 ± 0.002 a	ns
	Verrucomicrobia	1.206 ± 4.340 a	0.003 ± 0.003 a	0.001 ± 0.002 a	ns
	Betaproteobacteria	0.672 ± 1.301 a	0.212 ± 0.348 a	0.068 ± 0.065 a	ns
	Alphaproteobacteria	0.315 ± 0.514 a	0.111 ± 0.240 a	0.048 ± 0.036 a	ns
	Deltaproteobacteria	0.167 ± 0.601 a	0.000 ± 0.000 a	0.000 ± 0.000 a	ns
	Others	0.181 ± 0.487 a	0.044 ± 0.071 a	0.016 ± 0.015 a	ns
Rich sub-group	Firmicutes	10.534 ± 27.453 a	2.446 ± 6.010 a	3.359 ± 6.974 a	ns
	Gammaproteobacteria	0.449 ± 0.986 a	1.603 ± 5.764 a	0.050 ± 0.171 a	ns
Intermediate sub-group	**Firmicutes**	16.449 ± 24.662 **a**	58.065 ± 26.882 **b**	12.062 ± 19.679 **a**	2.24×10^{-5}
	Betaproteobacteria	0.146 ± 0.082 a	1.132 ± 3.562 a	0.146 ± 0.044 a	ns

Table S4. Properties of the bacterial microbiota association networks in different groups.

**Properties of the networks**	**NC**	**NEC**	**pdNEC**
Nodes[a]	1153	1119	1093
Edges[b]	2458	1453	1572
Average degree distribution[c]	66.81	50.527	56.016
Modularity[d]	0.506	0.512	0.518
Average clustering coefficient[e]	0.223	0.209	0.206
Average path length[f]	2.585	2.617	2.603

Modules	7	8	6

[a] Number of AVSs with the Spearman correlation |r| > 0.6 and *P*-value < 0.05. [b] Number of significant (*P*-value < 0.05) correlations between nodes. [c] The larger the average distribution, the more complex the network distribution. [d] Modularity > 0.4 suggested that the network has a modular structure.[e] How nodes were embedded in their neighborhood, and the degree to which nodes tend to cluster together. [f] The capability of the nodes to form highly connected communities.

Table S5. Classification and relative abundance of bacterial biomarkers.

No.	Phylum	Genus	subset	Mean RA	NC RA	NEC RA	pdNEC RA
	**Proteobacteria**			**12.057**			
1		Unassigned	rare	3.2192	0.0846	0.0737	9.4993
2		Unassigned	rare	2.7448	0.0399	0.0491	8.1454
3		Unassigned	rare	2.1995	0.0412	0.0431	6.5141
4		Unassigned	rare	2.0853	0.0432	0.0432	6.1697
5		Unassigned	rare	0.4483	0.0200	0.0029	1.3222
6		Unassigned	rare	0.3768	0.0227	0.0030	1.1047
7		Unassigned	rare	0.3084	0.0123	0.0152	0.8977
8		Unassigned	rare	0.2858	0.0206	0.0023	0.8344
9		Unassigned	rare	0.106	0.0045	0.0057	0.3078
10		Unassigned	rare	0.1035	0.0031	0.0052	0.3022
11		Unassigned	rare	0.0568	0.0012	0.0029	0.1662
12		Unassigned	rare	0.0239	0.0006	0.0012	0.0700
13		Unassigned	rare	0.0119	0.0009	0.0010	0.0337
14		Unassigned	rare	0.0073	0.0001	0.0003	0.0214
15		Unassigned	rare	0.0071	0.0006	0.0003	0.0202
16		Unassigned	rare	0.0054	0.0002	0.0002	0.0159
17		Unassigned	rare	0.0043	0.0000	0.0000	0.0128
18		Unassigned	rare	0.0042	0.0000	0.0004	0.0124
19		Unassigned	rare	0.0037	0.0004	0.0002	0.0104
20		Unassigned	rare	0.0031	0.0000	0.0002	0.0090
21		Unassigned	rare	0.0026	0.0000	0.0001	0.0076
22		Unassigned	rare	0.0019	0.0001	0.0000	0.0057
23		Unassigned	rare	0.0014	0.0000	0.0000	0.0042
24		Unassigned	rare	0.0012	0.0000	0.0000	0.0035
25		Unassigned	rare	0.0008	0.0000	0.0000	0.0023
26		*Sphingomonas*	rare	0.0002	0.0000	0.0001	0.0006
27		*Phenylobacterium*	rare	0.0013	0.0035	0.0003	0.0001
28		*Klebsiella*	rare	0.0162	0.0002	0.0005	0.0479
29		*Klebsiella*	rare	0.0035	0.0000	0.0000	0.0106
30		*Klebsiella*	rare	0.0009	0.0000	0.0000	0.0026
31		*Janthinobacterium*	rare	0.0019	0.0041	0.0007	0.0009
32		*Escherichia/Shigella*	rare	0.0028	0.0000	0.0035	0.0050

33		*Escherichia/Shigella*	rare	0.002	0.0001	0.0032	0.0026
34		*Delftia*	rare	0.0004	0.0001	0.0006	0.0005
35		*Curvibacter*	rare	0.0146	0.0000	0.0035	0.0050
	**Firmicutes**			**23.584**			
36		*Veillonella*	rare	0.5193	0.7520	0.3653	0.4408
37		*Veillonella*	rare	0.4825	0.0241	1.3220	0.1013
38		Unassigned	rare	0.0022	0.0055	0.0000	0.0011
39		Unassigned	intermediate	0.0053	0.0065	0.0054	0.0038
40		*Streptococcus*	rare	0.02			
41		*Streptococcus*	intermediate	0.787	1.5497	0.6447	0.1667
42		*Staphylococcus*	rare	0.0026	0.0029	0.0046	0.0001
43		*Staphylococcus*	rare	0.0014	0.0002	0.0039	0.0001
44		*Staphylococcus*	rare	0.0009	0.0001	0.0025	0.0000
45		*Lactobacillus*	rare	0.0048	0.0132	0.0002	0.0009
46		*Finegoldia*	rare	0.0288	0.0000	0.0822	0.0042
47		*Finegoldia*	rare	0.0141	0.0000	0.0415	0.0008
48		*Enterococcus*	rare	0.4503	0.1310	0.9447	0.2750
49		*Enterococcus*	rare	0.0207	0.0000	0.0606	0.0015
50		*Enterococcus*	rare	0.0171	0.0067	0.0408	0.0036
51		*Enterococcus*	rare	0.0162	0.0084	0.0370	0.0033
52		*Enterococcus*	rare	0.0158	0.0104	0.0340	0.0030
53		*Enterococcus*	rare	0.0133	0.0061	0.0310	0.0027
54		*Enterococcus*	rare	0.0004	0.0000	0.0012	0.0000
55		*Enterococcus*	rare	0.0004	0.0000	0.0011	0.0000
56		*Enterococcus*	intermediate	7.2181	1.6083	17.7204	2.3257
57		*Enterococcus*	intermediate	6.558	1.5672	16.0015	2.1051
58		*Enterococcus*	intermediate	4.9283	1.1685	12.1737	1.4427
59		*Enterococcus*	intermediate	2.4133	0.5669	5.9311	0.7417
60		*Clostridium_XlVa*	rare	0.0205	0.0001	0.0017	0.0597
61		*Anaerococcus*	rare	0.0287	0.0004	0.0857	0.0001
62		*Anaerococcus*	rare	0.0141	0.0000	0.0360	0.0064
	**Bacteroidetes**			**1.485**			
63		*Bacteroides*	rare	1.4588	4.2733	0.0027	0.1004
64		*Bacteroides*	rare	0.0261	0.0000	0.0000	0.0783
	**Actinobacteria**			**0.881**			
65		*Bifidobacterium*	rare	0.862	2.0306	0.0023	0.5530
66		*Arthrobacter*	rare	0.0112	0.0043	0.0232	0.0060
67		*Arthrobacter*	rare	0.0051	0.0014	0.0116	0.0022
68		*Arthrobacter*	rare	0.0026	0.0007	0.0058	0.0014
69		*Arthrobacter*	rare	0.0004	0.0000	0.0009	0.0002
70		*Arthrobacter*	rare	0.0002	0.0000	0.0007	0.0000

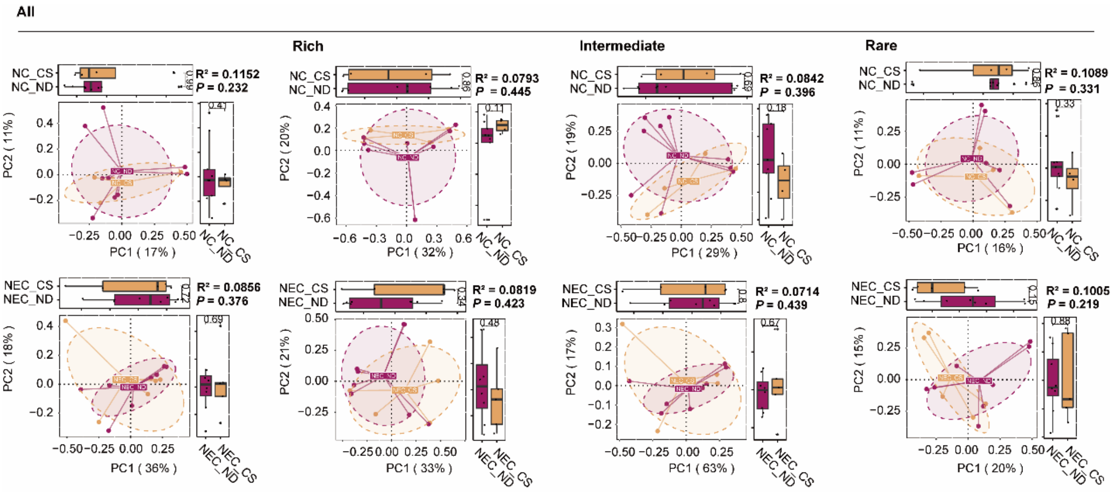

Fig. S1. Comparisons to the β-diversity distance and dissimilarity index of bacterial microbiota in nature delivery (ND) and caesarean section (CS) groups. Significant changes in beta diversity were calculated using PERMANOVA on the Bray-Curtis distance matrix. Lowercase letter represented significant differences of dissimilarity index between groups, based on Kruskal-Wallis test combined Wilcoxon tests.
